# Comparison of Antimicrobial Treatment Incidence Quantification Based on Detailed Field Data on Animal Level with the Standardized Methodology of the European Medicines Agency in Veal Calves, Switzerland, 2016–2018

**DOI:** 10.3390/antibiotics10070832

**Published:** 2021-07-08

**Authors:** Jens Becker, Mireille Meylan

**Affiliations:** Clinic for Ruminants, Vetsuisse-Faculty, University of Bern, Bremgartenstrasse 109a, 3012 Bern, Switzerland; mireille.meylan@vetsuisse.unibe.ch

**Keywords:** treatment intensity, antimicrobial use, standardization, agreement

## Abstract

Precise quantification of antimicrobial treatment incidence (TI) is crucial for benchmarking. Two widespread methods for treatment incidence quantification were compared for agreement. Field data were obtained from 38 veal farms from 2016 to 2018 (1905 calves, 1864 treatments). Calculation of TI_swiss_ for calves was based on detailed treatment records using pharmacokinetic values from the Swiss Veterinary Medicines Compendium. The method published by the European Medicines Agency was used to calculate TI in defined daily doses (TI_DDD_). For each calf and treatment, TI_swiss_ and TI_DDD_ were calculated on level of the antimicrobial class, drug, application route, and farm. The quotient (Q) of TI_swiss_ and TI_DDD_ was calculated. Divergence in results between the two methods of ≤25% was arbitrarily set as good agreement. The agreement between TI_swiss_ and TI_DDD_ was mostly good. On class level, good agreement was observed for treatments representing 71.5% of the TI_DDD,_ and 74.5% of the total TI_DDD_ on drug level. Poor agreement was mainly observed for tylosin and sulfadimidine. The agreement was better for parenteral than for oral treatments (81.6% vs. 72.3%). For practically orientated calculation on farm level, good agreement was observed (77.5% of the TI_DDD_). The TI_DDD_ method showed mostly good agreement, especially for parenteral treatments.

## 1. Introduction

Antimicrobial resistance (AMR) is likely to become an obstacle for human development, as antimicrobials may become ineffective for disease control and health care costs may rise considerably [1]. There is a large basis of evidence showing that antimicrobial use (AMU) is a major driver for the development of AMR in human and veterinary medicine through selection of resistant strains, which emphasizes the need to reduce AMU [2]. Different methods are used to quantify and compare AMU among countries, with the aim of developing strategies to monitor drug use and to identify excessive or inadequate use. Sales figures of antimicrobial therapeutic products have been published regularly for various countries for more than a decade [3,4,5,6]. However, an important limitation of the interpretation of sales figures is that antimicrobials cannot always be attributed to different animal species and production branches, and dose or duration of the treatments are rarely known. Thus, sales figures interpretation is difficult. Moreover, the amount of antimicrobials sold is not identical to the amount used, as, e.g., therapeutic products may expire before administration or packages may break. There is agreement that more detailed data than sales figures are needed to estimate AMU more precisely at the levels of the species, production branch, or farm [7].

Numerous methods and differing approaches have been developed to estimate AMU. However, comparison of the results of different methods often requires complicated conversion calculations or may be impossible. For example, the method for calculating the number of animal course doses is based on the summary of product characteristics (SPC, a legal document presenting the approved conditions of use and the properties of a drug [8]) and indicates the number of full-length treatments an animal was exposed to [9]. No direct comparison is possible with another method indicating the number of treatments per calves (NTPC) based on prescribed daily doses (PDD), even in the same country [10]. To use the NTPC method, very detailed data are necessary, including, e.g., the number of prescribed daily doses and an individual estimated weight for each treatment. Treatment incidence (TI) in animal daily doses (ADD) was given per calf-year in another study [11], and in the number of calves treated daily per 1000 calves at risk elsewhere [12]. Establishing a method which allows for comparison between studies and which requires limited data for calculation, which is easy to use and to interpret, and which applies to as many species and production branches as possible, is a major methodological challenge.

A method for TI quantification using defined daily doses (TI_DDD_) has been established by the European Medicines Agency [13,14]. It was developed based on the SPC from nine countries of the European Union [15], whereby a DDD is defined as the average estimated dose needed to treat one kilogram of the animal for the drug’s main indication for one day. The subsequent calculation of TI from the amount of drug used includes a standard weight for the typical animal to be treated, the observation period (i.e., the number of days the calves were observed, and the treatments were recorded), and the number of calves present. The unit of the result is the number of treatment days per animal, or per animal-year if a factor for extension to a year’s period is added to the formula. Values of DDD are provided by the EMA for most common drugs, for several species (cattle, swine, turkeys, and broilers), and administration routes (oral, parenteral; for cows additionally intramammary and intrauterine administration). This method allows for a direct result comparison among studies. However, there are limitations which may influence the results, e.g., the fact that different therapeutic products are used for a single drug in veterinary practice in Europe, and that their real potency, i.e., the actual dosages used as well as the weight of the animal at the time of treatment, may differ from the assumptions of the EMA based on average SPC values [11]. Therefore, the benefits of the method for comparing AMU are counterbalanced by reduced accuracy of AMU quantification. This may bias AMU reports and cause misinterpretation of the results. Nonetheless, for monitoring purposes, methods using DDD’s have been adopted by the World Health Organization and by national monitoring programs of the Netherlands and Scandinavian countries [2,4,5,16].

The aim of the present study was to investigate how precisely the calculated TI_DDD_ reflects the TI_swiss_ calculated based on indications for use from the Swiss Veterinary Medicines Compendium [17] for one species and one production branch, using detailed field treatment data on individual animal level from veal calves in Switzerland.

## 2. Material and Methods

### 2.1. Data Collection and Treatment Recording

Antimicrobial treatments (hereafter ‘treatments’) were recorded on 38 Swiss veal calf fattening farms between October 2016 and July 2018 in the frame of a previous study [18]. Briefly, farms were either intervention farms implementing a novel concept for veal calf fattening or control farms, and all calves were fattened according to the label IP-SUISSE which requires higher standards for animal welfare and sustainability than the Swiss legislation [19]. All components and procedures of the concept were approved by the competent authority (authorization number BE 71/16). Only the essential information for the present study is presented here. The novel concept was developed to mitigate the effects of the risk factors for increased AMU and mortality identified in previous studies [11,20]. The ‘outdoor veal calf’ concept is described in detail elsewhere [18]. The main hypothesis for that study was that AMU would be lower by at least 50% on intervention farms compared to controls. Antimicrobial use was assessed in detail during farm visits, which were performed at higher frequency than in other veal calf studies [12,21,22]. Each farm was followed for a minimum of twelve consecutive months and visited once monthly by one member of the study team (consisting of 3 veterinarians). A total of 535 farm visits were conducted. Treatments were recorded in detail by use of a specially customized booklet on each farm in accordance with statutory requirements (identification number of the treated animal(s), first and last administration date, name of therapeutic product, dosage, indication for treatment, administration route (oral vs. parenteral (i.e., intravenous, intramuscular or subcutaneous); and withdrawal period). Additionally, information on administration type (individual or group treatment), observed signs of disease, and treatment outcome were recorded. Only treatments with therapeutic products containing antimicrobials were recorded. Treatment modalities were determined at the farm veterinarians’ discretion, and the record booklets were filled in by the veterinarians with the farmers. The study team neither provided advice nor commented on the treatments. Dosages of the drugs were determined by farm veterinarians through estimation of the live body weight of the calves at the time of treatment. Diagnostic investigations such as culture of nasopharyngeal swabs or fluids obtained by bronchoalveolar lavage, and determination of susceptibility to antimicrobial drugs was at the discretion of the treating veterinarians; susceptibility testing was mostly not performed prior to treatment. A total of 1905 calves were enrolled, of which 731 received antimicrobial treatment (38.4%); a total of 1864 treatments were recorded. Treatment records were revised at each visit and incomplete records were completed with the farmer or the respective veterinarian, if necessary.

### 2.2. Treatment Incidence Quantification

The treatment incidences (TI_swiss_ and TI_DDD_) were calculated separately for each calf and each treatment. Treatments with therapeutic products containing two drugs were handled as two separate treatments in both methods. The unit of TI is the number of daily doses per calf (for TI_swiss_ and TI_DDD_) [13,14].

### 2.3. TI Quantification Based on Data from the Swiss Veterinary Medicines Compendium (TI_swiss_)

All used drugs were registered with the Swiss authority for licensing therapeutic products (Swissmedic) [23]. Information on the pharmacokinetics of each product were available online (Swiss Veterinary Medicines Compendium, https://www.vetpharm.uzh.ch/tak/clinidoc.htm, accessed on 2 May 2020) [17]), and treatment according to the recommended dose for the weight of the animal as estimated by the treating veterinarian was assumed. The maintenance period of a drug was defined as the period (in days) during which the minimal concentration required for successful treatment was obtained. Drugs were administered either once or several times with varying intervals between applications. Oral and parenteral route were used for application. Therefore, the following categories of administration modalities were differentiated:

(A) Daily oral or parenteral drug administration: for drugs which were administered orally or parenterally once daily during several consecutive days, the maintenance period was equivalent to the number of days the drug was administered.

(B) Repeated parenteral administration at two-day intervals: for drugs which were administered parenterally at two-day intervals (i.e., drugs with a maintenance period of two days), each administration day was counted as two treatment days.

(C) Single administration of long-acting preparations: for drugs which were administered once, the maintenance period indicated in the Swiss Veterinary Medicines Compendium was used. If this maintenance period was indicated to be a range of days (for example 5–7 days), the average maintenance period was used (i.e., 6 days).

(D) Repeated administration of drugs with overlapping maintenance periods: for drugs which were prescribed to be administered at a certain interval but of which the maintenance period exceeds this interval, TI_swiss_ was obtained as follows: number of applications * interval between applications + (maintenance period-interval between applications). Thus, for a drug with a maintenance period of 2.5 days which was administered twice at an interval of two days, the TI_swiss_ is 4.5 [2 × 2 + (2.5 − 2) = 4.5].

(E) Drugs without indication of a maintenance period: for tulathromycin, no maintenance period is indicated in the Swiss Veterinary Medicines Compendium. It was arbitrarily set to be 10 days after single parenteral administration of 2.5 mg/kg based on the following observations: after single application of 2.5 mg/kg plasma concentrations were >20 ng/mL for up to 12 days [24,25]. Pulmonary concentrations (in pulmonary epithelial lining fluid) were estimated to be approximatively 100 times higher (i.e., 2 µg/mL) [26]. The minimal inhibitory concentrations were 1–4 µg/mL for Pasteurellaceae [27]. Thus, pulmonary concentrations met the average minimum inhibitory concentration for approximatively 10 days.

### 2.4. Treatment Incidence Quantification with the EMA Method (TI_DDD_)

The same dataset (as for the calculation of TI_swiss_) was used. Values for DDD were available for all drugs used and their respective administration routes. For calculation, the following Formula (1) was used:

(1) $${\mathrm{TI}}_{\mathrm{DDD}}=\frac{total\;amount\;of\;drug\;administered\;\left(mg\right)}{DDD\;\left(\frac{mg}{kg}\right)*\;observation\;period\;\left(days\right)*number\;of\;calves\;present*\;standard\;weight\;\left(kg\right)}$$

In contrast to the calculation of TI_swiss_, the standard weight at treatment of 80 kg defined by the EMA was applied here for all calves and all treatments [13,14].

### 2.5. Statistical Analyses

For every treatment, a quotient (Q) of TI_swiss_ and TI_DDD_ (Q = TI_swiss_/TI_DDD_) was calculated. When Q = 1, TI_swiss_ equals TI_DDD_; when Q < 1, the estimation of TI by TI_swiss_ is lower than the one by TI_DDD_; and when Q > 1, the estimation of TI by TI_swiss_ is higher than the one by TI_DDD_. Treatments were grouped by antimicrobial class, drug, application route, and farm ID. Subsequently, the number of treatments, the sum of TI_swiss_, the sum of TI_DDD_, and the percentage of the total TI per group were calculated. For each group, the median of Q and the interquartile range (IQR) of Q were calculated. For antimicrobial classes, drugs, application routes or farms, respectively, where Q values indicated a discrepancy between TI_swiss_ and TI_DDD_ ≤ 0.25 (25%), the methods were arbitrarily considered to be in good agreement with each other. In observer reliability statistics, a discrepancy up to 0.20 is still considered ‘almost perfect’ agreement, and a discrepancy up to 0.40 as ‘substantial’ agreement [28,29,30]. Drugs used less than 10 times were excluded from further analyses, regardless of the TI. Additionally, EMA DDD values were directly compared with ‘daily doses’ calculated based on the extensive dataset of TI_swiss_, as described above. For this calculation of ‘daily doses’, the equation for TI_DDD_ calculation was rearranged by solving for the position of the factor ‘DDD’. This factor was renamed ‘daily dose’, and values were obtained by inserting the required values ‘amount of drug used’, ‘observation period’, and ‘number of calves present’. The factor ‘standard weight’ was replaced by the estimated mean live body weight at treatment (separately for oral and parenteral treatments; 111.0 kg for oral and 124.1 kg for parenteral treatments). These standardized weights were calculated based on an assumed start weight at the beginning of the fattening period of 72.1 kg, a constant weight gain throughout the fattening period, and the average treatment time point as described elsewhere (mean weight and age at treatment ± standard deviation 119.3 kg ± 43.4; 72.1 days ± 29.1) [18]. Calculations were performed using Microsoft Excel^®^ (Microsoft, Redmont, WA, USA), and ‘R’ Version 3.5.1 (R Core Team 2020, R Foundation for Statistical Computing, Vienna, Austria).

## 3. Results

The most frequently used antimicrobial classes by TI_DDD_ were tetracyclines (45.6%), penicillins (19.1%), and macrolides (12.4%). The most frequently used antimicrobial classes by TI_swiss_ were tetracyclines (32.5%), macrolides (25.5%) and sulfonamides (20.2%).

Antimicrobial treatments representing 73.3% of the TI_DDD_ were administered orally through the feed (mixed with milk or milk replacer), whereas 26.7% were administered parenterally through either intravenous, intramuscular or subcutaneous injection. Antimicrobial treatments representing 71.5% of the TI_DDD_ were administered as group treatments, and 28.5% as individual treatments. Group treatments were considerably more frequently administered orally than parenterally (95.9% vs. 4.1% of the TI_DDD_). Individual treatments were mainly administered parenterally (83.4% vs. 16.6% of the TI_DDD_).

Grouped by antimicrobial class, treatments corresponding to 71.5% (all treatments) differed by ≤ 25% between TI_swiss_ and TI_DDD_ (Table 1).

For the comparison of TI_swiss_ and TI_DDD_ on drug level, treatments with the following drugs were excluded due to low numbers of observations (number of observations): neomycin, spectinomycin, lincomycin, enrofloxacin, sulfamethoxypyridazine (1); procaine benzylpenicillin/benzathin, cefquinome, sulfadoxine (2); amoxicillin/clavulanic acid, sulfadiazine, sulfadoxine (3); ceftiofour (7). Of the remaining 1837 treatments, the most frequently used drugs by TI_DDD_ were chlortetracycline (28.4%), amoxicillin (15.4%), and oxytetracycline (8.7%). The most frequently used drugs by TI_swiss_ were chlortetracycline (21.5%), sulfadimidine (17.1%), and tylosin (14.1%). Grouped by drug, treatments corresponding to 74.5% of the total TI_DDD_ differed by ≤25% (Table 2).

The most frequently drugs used for oral application were chlortetracycline (38.4 % of the TI_DDD_), doxycycline (11.6%), and sulfadimidine (11.3%), and treatments corresponding to 72.3% of the total TI_DDD_ differed by ≤25% (Table 3).

The most frequently drugs used for parenteral application were oxytetracycline (33.8% of the TI_DDD_), tulathromycin (19.5%), and procaine benzylpenicillin (14.2%). Of those, treatments corresponding to 81.6% of the total TI_DDD_ differed by ≤25% (Table 4).

Of 38 participating farms, the three farms with the highest TI by TI_DDD_ accounted for 13.3%, 12.3%, and 11.4% of the total TI_DDD_. Two of those were part of the three farms with the highest TI by TI_swiss_, which accounted for 15.0%, 12.9%, and 11.4% of the total TI_swiss_. Grouped by farm ID, treatments corresponding to 75.5% of the total TI_DDD_ values differed by ≤25% (Table 5).

Table 6 presents the EMA DDD values as well as the hypothetical ‘daily doses’ for TI_swiss_. According to the previous results, the newly calculated ‘daily doses’ are closely comparable for most drugs, except for sulfonamides, most macrolides and streptomycin. There is overall better agreement for parenteral DDD’s with ‘daily doses’ values than for oral values.

## 4. Discussion

Overall, a good agreement between the two methods for TI calculation was observed. It is improbable to achieve a full agreement (100%) between the two methods for TI quantification as both are based on a number of assumptions. The TI_DDD_ method was developed to allow for standardization of TI with a multitude of products licensed in different countries. When using the treatment record dataset of the present study, the method provided good standardization. Limited deviations to the ‘true’ TI may be acceptable, as the achieved standardization outweighs the negative effects of deviations. Calculating and reporting standardized TI is crucial for benchmarking, and allows for comparison of TI between production branches, farms, and countries as well as over time. This way, it is possible, e.g., to classify farmers as high, intermediate, or low users of antimicrobials in the frame of monitoring and prevention programs.

Discrepancy between TI values was quantified similarly to another study by calculation of the quotient Q [31]. We considered a divergence of up to 25% between TI_swiss_ and TI_DDD_ to represent a good agreement. This threshold was arbitrarily set yet follows the principles of classical correlation statistics. The threshold may be a subject of discussion. When considering the range of countries and the number of therapeutic products for which the TI_DDD_ method was developed, we suggest that this threshold is suitable, and the TI_DDD_ method can be considered to serve its purpose. If the threshold was extended to ≤30%, the percentage of treatments for which both methods provide agreeing results would increase to a small extent only (by 1.9% due to treatments of the class of fluoroquinolones and 5.8% due to one farm, respectively). In contrast, if the threshold value was set to ≤20%, the agreement between the two methods would be moderately to considerably lower. This would almost exclusively be due to a lower agreement for chlortetracycline (−28.4% and −38.4% when grouped by class and for oral treatments, respectively), although the agreement for the antimicrobial class ‘tetracyclines’ as a whole would not be affected and the agreement for oxytetracycline would still be classified as ‘good’. The lower agreement would also be due to reclassification of treatments with the class ‘penicillins’ (−19.1%). The agreement for amoxicillin would still be classified as ‘good’, but not for procaine benzylpenicillin. Tetracylines were frequently used in the calves enrolled in this study. Therefore, a shift from ‘agreement’ to ‘non-agreement’ of one drug belonging to the tetracycline class may have a great impact if analyzed on drug level, and no or little impact if analyzed on the level of the antimicrobial class and for parenteral treatments only. Using the most practical approach where agreement was calculated on farm level, i.e., on a set of treatments that had been used in real life, a lower threshold of ≤20% would entail a minimal change (0.8% of the total TI_DDD_ would no longer be classified as ‘good agreement’). This shows that both methods provide similar results especially when used on class level and for the purpose of TI calculation of farm level treatment records. Alternatively, TI calculation may also be conducted with prescribed amounts of antimicrobials, implying the risk of those not reflecting accurately the truly administered amounts [32].

All methods that are used for TI calculation are based on assumptions to varying degrees. This includes the ‘real’ TI where values of the Swiss Veterinary Medicines Compendium are used for calculation. Correspondingly, none of the methods should be regarded as gold standard and be used as reference. By agreeing on one method for TI quantification in the field, the same assumptions would be made in all calculations. This would increase comparability between countries and production types.

In many veal calf studies, farm visits are conducted at intervals of several months [22]. We conducted labor-intensive monthly farm visits aimed principally at following up closely on the treatment history of the farms. By revising each of close to 2000 treatment records one month or less after administration and cross-checking animal ear tag numbers, we achieved good data quality on calf level. Clarifications could be addressed with the farmer or the farm veterinarian within short delays, if necessary. This way, we were able to eliminate as many inaccuracies as possible before losing track on treatment modalities as may be the case when collecting data retrospectively. In contrast to a similar study in swine [33], treatments were recorded prospectively to avoid loss of information due to incomplete data storage.

For correct TI_DDD_ calculation, the observation period is needed [13,14]. By using data from the animal traffic database and slaughter data, we obtained the exact observation period for each calf. Therefore, we are confident that the data quality was good and an accurate estimation of TI_DDD_ was obtained which qualified for comparison.

The results of this study are restricted to the production branch of veal calves. It would be useful to perform similar studies using detailed treatment record datasets from populations of adult cattle, or other species the EMA provides DDD values for. According to the species or production branch, different DDD values and standard weights at treatment apply. Therefore, it is not possible to estimate the agreement of the methods for those species based on the present results.

The repertoire of drugs used in the present study as well as their distribution are in accordance with the results of another recent study conducted in a different subset of Swiss veal farms [34]. This underlines that our dataset represents commonly applied drugs. In the present study, a few drugs were excluded to avoid drawing conclusions on an entire antimicrobial class from a low number of observed treatments. In regard to highest priority critically important antimicrobials (HPCIA), the use of third and fourth generation cephalosporins was very low and such treatments were excluded due to the low numbers of observations. Therefore, the two methods of TI quantification cannot be compared for this antimicrobial class. Agreement between TI_swiss_ and TI_DDD_ for fluoroquinolones as a group was below the limit for good agreement with a median Q value of 0.72, with large differences between the two drugs analyzed (median Q = 0.90 for marbofloxacin and 0.47 for danofloxacin). The guidelines of the IP-SUISSE label applied in all farms of the study require that macrolides, quinolones and third and fourth generation cephalosporins may only be used in exceptional cases following a written justification by the farm veterinarian. This may have contributed to a limited use of cephalosporins; however, their use was also low in another study where mostly non-IP-SUISSE farms were followed, suggesting that there are sufficient alternatives to replace cephalosporins in practice [21]. Furthermore, the use of fluoroquinolones, which are used for individual treatments as cephalosporins higher generation, was distinctly higher with 6–7% (depending on the quantification method) of parenteral treatments. Likewise, the use of macrolides was high for oral and for parenteral treatments, and thus label restrictions obviously did not halt the use of all HPCIA in study farms.

In addition to the methodological comparison of TI_swiss_ and TI_DDD_, we calculated ‘daily doses’ based on the treatment data of the present study to illustrate the agreement of the methods on that level (Table 6). We explicitly did not aim at suggesting alternative defined daily doses, we took advantage of the fact that, in contrast to other field studies, it was possible to calculate ‘daily doses’ from calf-level treatment data because the exact observation period was available This direct comparison did not reveal a trend in any direction (consistently higher or lower TI values with one method), which underlines the specificities of each drug and thus the fact that each drug must be considered separately.

Besides a general good agreement, a pronounced discrepancy was observed for few antimicrobial drugs and classes. Mainly, the discrepancy between TI_swiss_ and TI_DDD_ for tylosin and sulfonamides negatively influenced the overall agreement of the two methods. Therapeutic products containing tylosin alongside other antimicrobials are licensed with lower daily doses in Switzerland compared with EMA values, as a synergistic effect of the different antimicrobials to certain bacteria had been suggested by some (despite a lack of evidence to support this statement) [13,31,32]. Therefore, lower oral daily doses for tylosin are indicated in the Swiss Veterinary Medicines Compendium than in the EMA method. This may explain why the discrepancy in tylosin TI’s between the two methods exceeds 25% by far. For sulfonamides, many different products are available. The EMA provides separate DDD values for sulfonamides and trimethoprim when used alone and in combination. To improve the agreement for sulfonamides, specific DDD values would have to be assigned to each licensed therapeutic product currently on sale. This approach would lead to a more precise estimation of TI_DDD_. However, the large number of licensed commercial therapeutic products would make the determination of DDD values for each product excessively labor- and cost-intensive.

Especially for the most practical approach where treatments were grouped on farm level, 77.5% of the total TI_DDD_ values represented treatments which diverged by less than 25% from TI_swiss_ values. This further supports that the standardized TI_DDD_ method is useful for TI estimation. Frequently, therapeutic products used for metaphylactic treatment contain two or more drugs (for example SK 60 ad. us. vet ^®^, Biokema SA, 1023 Crissier, CH) containing spiramycine and chlortetracycline), leading to simultaneous administration of the two drugs. For this product, median Q values were above and below 1.00 (1.94 and 0.78 for spiramycin and chlortetracycline, respectively); thus, this is one example where the two components of a product equal out each other to a certain extent. This is, however, not applicable to all products containing more than one drug.

Despite the well-known limitations of the EMA method [13,14], the availability of one single standardized method providing results that can be compared within and among countries and production branches in the course of years is of utmost importance. Especially for the veal calf production branch which was observed to use relatively high amounts of antimicrobials, accurate quantification methods are needed to document the evolution of AMU over time.

## 5. Conclusions

In summary, reporting AMU by using the TI_DDD_ method is reasonably accurate for most investigated drugs in veal calf production branch based on our dataset. Trading off the information needed to calculate TI_DDD_ with the output it generates, this method provides an efficient way to obtain reliable and comparable results.

## Figures and Tables

**Table 1:** Antimicrobial use (grouped by antimicrobial class) in 38 veal operations in Switzerland (*n* = 1854 treatments). For each antimicrobial class, the number of treatments, the sum of treatment days, and the respective percentage of all treatments are given as treatment incidence (TI) based on the summary of product characteristics of the Swiss Veterinary Medicines Compendium (TI_swiss_) ^1^ or on defined daily doses (TI_DDD_) according to the European Medicines Agency ^2^, respectively. To compare the two TI’s, the quotient Q (Q = TI_swiss_/TI_DDD_) was calculated for each treatment, and the median value of Q for all treatments with a given drug as well as its interquartile range (IQR_Q_) are given. Antimicrobial classes for which the agreement between TI_swiss_ and TI_DDD_ was good (maximal discrepancy ≤ 25%) are highlighted in grey.

Agreement between TI_swiss_ and TI_DDD_ Methods	Antimicrobial Class	n ^3^	Sum of TI_swiss_ (Days)	% of Total TI_swiss_^4^	Sum of TI_DDD_ (Days)	% of Total TI_DDD_^5^	Median of Q	IQR of Q
Discrepancy > 25% (TI_DDD_ < TI_swiss_)	Macrolides	372	3157	25.5	1548.91	12.4	1.94	1.56–7.42
	Sulfonamides	343	2499	20.2	1351.75	10.8	1.87	1.42–2.33
TI_swiss_ in good agreement with TI_DDD_ (maximal discrepancy ≤ 25%)	Phenicols	10	43.26	0.4	36.71	0.3	1.16	1.16–1.16
	Amino- glycosides	32	150	1.2	140.87	1.1	1.14	0.80–2.07
	Florfenicols	101	330.93	2.7	439.64	3.5	1.07	0.57–1.16
	Tetracyclines	593	4017	32.5	5682.93	45.6	0.80	0.63–0.92
	Penicillins	292	1638	13.3	2383.37	19.1	0.76	0.49–0.95
Discrepancy > 25% (TI_DDD_ > TI_swiss_)	Fluoro- quinolones	51	143	1.2	238.53	1.9	0.72	0.48–1.74
	Diamino- pyrimidins	60	379	3.1	636.43	5.1	0.60	0.58–0.60
Sum ^6^		1854	12,357.19	100.1	12,459.14	99.8		
Sum		1079	6322.19	51.3	8922.05	71.5		

^1^ Tierarzneimittelkompendium der Schweiz, Institute of Pharmacology and Toxicology, Vetsuisse Faculty Zürich, Switzerland, [17]. ^2^ Defined daily doses for animals (DDDvet) and defined course doses for animals (DCDvet): European Surveillance of Veterinary Antimicrobial Consumption (ESVAC), and Revised ESVAC reflection paper on collecting data on consumption of antimicrobial agents per animal species, on technical units of measurement and indicators for reporting consumption of antimicrobial agents in animals [13,14]. ^3^ Number of antimicrobial treatments containing a drug of the respective class. ^4^ The percentage of the treatment incidence (TI) on the total TI is given for each antimicrobial class and was calculated using the values of the Swiss Veterinary Medicines Compendium. ^5^ The percentage of the TI on the total TI is given for each antimicrobial class and was calculated using the values of the European Medicines Agency ^6^ Sum of respective parameters; sum of the treatments for which TI_swiss_ and TI_DDD_ are in good agreement (highlighted in grey).

**Table 2:** Antimicrobial use (grouped by drug) in 38 veal operations in Switzerland (*n* = 1837 treatments). For each drug, the number of treatments, the sum of treatment days, and the respective percentage of all treatments are given as treatment incidence (TI) based on the summary of product characteristics from the Swiss Veterinary Medicines Compendium ^1^ (TI_swiss_) or on defined daily doses (TI_DDD_) according to the European Medicines Agency ^2^, respectively. To compare the two TI’s, the quotient Q (Q = TI_swiss_/TI_DDD_) was calculated for each treatment, and the median value of Q for all treatments with a given drug as well as its interquartile range (IQR of Q) are given. Drugs for which the agreement between TI_swiss_ and TI_DDD_ was good (maximal discrepancy ≤ 25%) are highlighted in grey.

Agreement between TI_swiss_ and TI_DDD_ Methods	Drug	Antimicrobial Class	n ^3^	Sum of TI_swiss_ (Days)	% of Total TI_swiss_^4^	Sum of TI_DDD_ (Days)	% of Total TI_DDD_^5^	Median of Q	IQR of Q
Discrepancy > 25% (TI_DDD_ < TI_swiss_)	Tylosin	Macrolides	222	1735	14.1	368.64	3.0	7.39	5.47–8.04
	Spiramycine	Macrolides	91	917	7.4	516.16	4.2	1.94	1.94–1.94
	Sulfadimidine	Sulfonamides	278	2109	17.1	1037.98	8.4	1.89	1.50–2.69
TI_swiss_ in good agreement with TI_DDD_ (maximal discrepancy ≤ 25%)	Phthalylsulfathiazole	Sulfonamides	56	370	3.0	301.49	2.4	1.25	1.20–1.25
	Tilmicosin	Macrolides	13	39	0.3	37.14	0.3	1.20	0.96–1.20
	Tulathromycin	Macrolides	46	466	3.8	626.97	5.0	1.20	0.78–1.20
	Florfenicol	Phenicols	10	43.26	0.4	36.71	0.3	1.16	1.16–1.16
	Dehydro- streptomycin	Aminoglycosides	30	145	1.2	138.82	1.1	1.07	0.80–1.90
	Florfenicol	Florfenicols	101	330.93	2.7	439.64	3.5	1.07	0.57–1.16
	Marbofloxacin	Fluoroquinolones	31	80	0.6	97.33	0.8	0.90	0.72–1.80
	Oxy- tetracycline	Tetracyclines	207	813	6.6	1086.86	8.7	0.87	0.52–1.14
	Amoxicillin	Penicillins	223	1389.5	11.3	1910.6	15.4	0.82	0.64–0.95
	Doxycycline	Tetracyclines	77	558	4.5	1069.52	8.6	0.80	0.80–0.80
	Chlor- tetracycline	Tetracyclines	309	2646	21.5	3526.55	28.4	0.78	0.63–0.92
Discrepancy > 25% (TI_DDD_ > TI_swiss_)	Trimethoprim	Diamino-pyrimidins	60	379	3.1	636.43	5.1	0.60	0.58–0.60
	Procaine benzylpenicillin	Penicillins	64	232	1.9	456.08	3.7	0.49	0.35–0.75
	Danofloxacin	Fluoroquinolones	19	61	0.5	138.22	1.1	0.47	0.34–0.63
Sum ^6^			1837	12313.69	100	12425.14	100		
Sum			1103	6880.69	55.9	9271.63	74.5		

^1^ Tierarzneimittelkompendium der Schweiz, Institute of Pharmacology and Toxicology, Vetsuisse Faculty Zürich, Switzerland, [17]. ^2^ Defined daily doses for animals (DDDvet) and defined course doses for animals (DCDvet): European Surveillance of Veterinary Antimicrobial Consumption (ESVAC), and Revised ESVAC reflection paper on collecting data on consumption of antimicrobial agents per animal species, on technical units of measurement and indicators for reporting consumption of antimicrobial agents in animals [13,14]. ^3^ Number of antimicrobial treatments containing the respective drug. ^4^ The percentage of the treatment incidence (TI) on the total TI is given for each drug and was calculated using the values of the Swiss Veterinary Medicines Compendium. ^5^ The percentage of the TI on the total TI is given for each drug and was calculated using the values of the European Medicines Agency. ^6^ Sum of respective parameters; sum of the treatments for which TI_swiss_ and TI_DDD_ are in good agreement (highlighted in grey).

**Table 3:** Antimicrobial use (oral treatments only, *n* = 1279) in 38 veal operations in Switzerland from October 2016 to July 2018. For each drug, the number of treatments, the sum of treatment days, and the respective percentage of all treatments are given as treatment incidence (TI) based on the summary of product characteristics of the Swiss Veterinary Medicines Compendium (TI_swiss_) ^1^ and on defined daily doses (TI_DDD_) according to the European Medicines Agency ^2^, respectively. To compare the two TI’s, the quotient Q (Q = TI_swiss_/TI_DDD_) was calculated for each treatment, and the median value of Q for all treatments with a given drug as well as its interquartile range (IQR_Q_) are given. Drugs for which the agreement between TI_swiss_ and TI_DDD_ was good (maximal discrepancy ≤ 25%) are highlighted in grey.

Agreement between TI_swiss_ and TI_DDD_ Methods	Drug	Antimircrobial Class	n ^3^	Sum of TI_swiss_ (Days)	% of Total TI_swiss_^4^	Sum of TI_DDD_ (Days)	% of Total TI_DDD_^5^	Median of Q	IQR of Q
Discrepancy > 25% (TI_DDD_ < TI_swiss_)	Tylosin	Macrolides	218	1729	17.4	361.72	3.9	7.39	5.47–8.04
	Spiramycine	Macrolides	91	917	9.2	516.16	5.6	1.94	1.94–1.94
	Sulfadimidine	Sulfonamides	276	2105	21.1	1035.8	11.3	1.89	1.50–2.69
TI_swiss_ in good agreement with TI_DDD_ (maximal discrepancy ≤ 25%)	Phthalyl- sulfathiazole	Sulfonamides	56	370	3.7	301.49	3.3	1.25	1.20–1.25
	Amoxicillin	Penicillins	197	1267.5	12.7	1749.05	19	0.82	0.64–0.95
	Doxycycline	Tetracyclines	77	558	5.6	1069.52	11.6	0.80	0.80–0.80
	Chlor- tetracycline	Tetracyclines	309	2646	26.6	3526.55	38.4	0.78	0.63–0.92
Discrepancy > 25% (TI_DDD_ > TI_swiss_)	Trimethoprim	Diaminopyrimidins	55	368	3.7	622.67	6.8	0.60	0.58–0.60
Sum ^6^			1279	9960.5	100	9182.96	99.9		
Sum			639	4841.5	48.6	6646.61	72.3		

^1^ Tierarzneimittelkompendium der Schweiz, Institute of Pharmacology and Toxicology, Vetsuisse Faculty Zürich, Switzerland, [17]. ^2^ Defined daily doses for animals (DDDvet) and defined course doses for animals (DCDvet): European Surveillance of Veterinary Antimicrobial Consumption (ESVAC), and Revised ESVAC reflection paper on collecting data on consumption of antimicrobial agents per animal species, on technical units of measurement and indicators for reporting consumption of antimicrobial agents in animals [13,14]. ^3^ Number of antimicrobial treatments containing the respective drug. ^4^ The percentage of the treatment incidence (TI) on the total TI is given for each drug and was calculated using the values of the Swiss Veterinary Medicines Compendium. ^5^ The percentage of the TI on the total TI is given for each drug and was calculated using the values of the European Medicines Agency. ^6^ Sum of respective parameter; sum of the treatment for which TI_swiss_ and TI_DDD_ are in good agreement (highlighted in grey).

**Table 4:** Antimicrobial use (parenteral treatments only, *n* = 547) in 38 veal operations in Switzerland from October 2016 to July 2018. For each drug, the number of treatments, the sum of treatment days, and the respective percentage of all treatments are given as treatment incidence (TI) based on the summary of product characteristics of the Swiss Veterinary Medicines Compendium (TI_swiss_) ^1^ and on defined daily doses (TI_DDD_) according to the European Medicines Agency ^2^, respectively. To compare the two TI’s, the quotient Q (Q = TI_swiss_/TI_DDD_) was calculated for each treatment, and the median value of Q for all treatments with a given drug as well as its interquartile range (IQR_Q_) are given. Drugs for which the agreement between TI_swiss_ and TI_DDD_ was good (maximal discrepancy ≤ 25%) are highlighted in grey.

Agreement between TI_swiss_ and TI_DDD_ Methods	Drug	Antimircrobial Class	n ^3^	Sum of TI_swiss_ (Days)	% of Total TI_swiss_^4^	Sum of TI_DDD_ (Days)	% of Total TI_DDD_^5^	Median of Q	IQR of Q
TI_swiss_ in good agreement with TI_DDD_ (maximal discrepancy ≤ 25%)	Tilmicosin	Macrolides	13	39	1.7	37.14	1.2	1.20	0.96–1.20
	Tulathro- mycin	Macrolides	46	466	20	626.97	19.5	1.20	0.78–1.20
	Florfenicol	Phenicols	10	43.26	1.9	36.71	1.1	1.16	1.16–1.16
	Dehydro- streptomcin	Aminoglycosides	30	145	6.2	138.82	4.3	1.07	0.80–1.90
	Florfenicol	Florfenicols	101	330.93	14.2	439.64	13.7	1.07	0.57–1.16
	Marbo- floxacin	Fluoroquinolones	31	80	3.4	97.33	3	0.90	0.72–1.80
Discrepancy > 25% (TI_DDD_ > TI_swiss_)	Amoxicillin	Penicillins	26	122	5.2	161.55	5	0.89	0.68–0.89
	Oxy- tetracycline	Tetracyclines	207	813	34.9	1086.86	33.8	0.87	0.52–1.14
	Procaine benzylpenicillin	Penicillins	64	232	9.9	456.08	14.2	0.49	0.35–0.76
	Danofloxacin	Fluoroquinolones	19	61	2.6	138.22	4.3	0.47	0.34–0.63
Sum ^6^			547	2332.19	100	3219.32	100.1		
Sum			464	2039.19	87.5	2625.02	81.6		

^1^ Tierarzneimittelkompendium der Schweiz, Institute of Pharmacology and Toxicology, Vetsuisse Faculty Zürich, Switzerland, [17]. ^2^ Defined daily doses for animals (DDDvet) and defined course doses for animals (DCDvet): European Surveillance of Veterinary Antimicrobial Consumption (ESVAC), and Revised ESVAC reflection paper on collecting data on consumption of antimicrobial agents per animal species, on technical units of measurement and indicators for reporting consumption of antimicrobial agents in animals [13,14]. ^3^ Number of antimicrobial treatments containing the respective drug. ^4^ The percentage of the treatment incidence (TI) on the total TI is given for each drug and was calculated using the values of the Swiss Veterinary Medicines Compendium. ^5^ The percentage of the TI on the total TI is given for each drug and was calculated using the values of the European Medicines Agency. ^6^ Sum of respective parameters; sum for the treatment for which TI_swiss_ and TI_DDD_ are in good agreement (highlighted in grey).

**Table 5:** Antimicrobial use (grouped by farm ID) in 38 veal operations in Switzerland from October 2016 to July 2018 (*n* = 1838 treatments). For each farm, the number of treatments, the sum of treatment days, and the respective percentage of all treatments are given as treatment incidence (TI) based on the summary of product characteristics of the Swiss Veterinary Medicines Compendium (TI_swiss_) ^1^ and on defined daily doses (TI_DDD_) according to the European Medicines Agency ^2^, respectively. To compare both TI’s, for each underlying treatment, the quotient Q (Q = TI_swiss_/TI_DDD_) was calculated and the median of Q as well as its interquartile range (IQR_Q_) are given. Farms for which TI_swiss_ was in accordance with TI_DDD_ (max. under/overestimation ≤ 25%) are highlighted in grey.

Agreement between TI_swiss_ and TI_DDD_ Methods	Farm ID	n ^3^	Sum of TI_swiss_ (Days)	% of Total TI_swiss_^4^	Sum of TI_DDD_ (Days)	% of Total TI_DDD_^5^	Median of Q	IQR of Q
Discrepancy > 25% (TI_DDD_ < TI_swiss_)	23	44	292.75	2.4	103.09	0.8	7.00	2.35–27.33
	24	19	93	0.8	49.55	0.4	2.00	1.73–2.67
	34	22	102.15	0.8	77.9	0.6	1.78	0.80–2.17
	3	46	256.41	2.1	272.98	2.2	1.30	0.92–1.80
	35	101	756	6.2	716.56	5.8	1.30	0.63–4.98
TI_swiss_ in good agreement with TI_DDD_ (maximal discrepancy ≤ 25%)	21	221	1575	12.9	1630.22	13.3	1.20	0.80–1.25
	18	17	41	0.3	33.28	0.3	1.17	0.65–3.50
	27	152	1400	11.4	1180.39	9.6	1.16	0.92–1.94
	25	230	1835.21	15.0	1397.21	11.4	1.14	0.89–2.69
	32	41	259	2.1	253.25	2.1	1.14	0.95–1.14
	33	19	44.5	0.4	46.62	0.4	1.08	0.87–1.08
	36	130	858	7.0	709.91	5.8	1.05	0.80–2.06
	37	64	431	3.5	352.17	2.9	0.96	0.85–2.06
	9	19	113.5	0.9	93.76	0.8	0.93	0.78–2.33
	38	165	711.56	5.8	825.21	6.7	0.90	0.63–1.89
	6	22	73.76	0.6	91.95	0.8	0.87	0.57–1.11
	17	11	34	0.3	33.31	0.3	0.87	0.56–16.21
	28	19	108.5	0.9	145.31	1.2	0.87	0.64–0.87
	31	70	494	4.0	558.5	4.6	0.87	0.61–1.30
	22	23	220	1.8	293.51	2.4	0.82	0.71–0.82
	26	44	161.5	1.3	226.45	1.8	0.81	0.54–1.30
	29	176	1380.63	11.3	1508.42	12.3	0.80	0.64–1.90
	16	16	68	0.6	94.89	0.8	0.77	0.63–0.98
Overestimation of TI by >25%	20	14	59.89	0.5	99.02	0.8	0.66	0.46–0.94
	39	67	521.82	4.3	602.72	4.9	0.63	0.47–1.40
	5	24	143.63	1.2	378.22	3.1	0.52	0.32–0.80
	14	15	42.5	0.3	92.68	0.8	0.42	0.35–0.52
	15	13	53.5	0.4	137.16	1.1	0.38	0.38–0.38
	40	23	66.25	0.5	124.22	1.0	0.35	0.35–0.89
	19	11	46.63	0.4	128.38	1.0	0.34	0.34–0.37
Sum ^6^		1838	12,243.69	100	12,256.84	100		
Sum		1439	9809.16	80.1	9474.36	77.5		

^1^ Tierarzneimittelkompendium der Schweiz, Institute of Pharmacology and Toxicology, Vetsuisse Faculty Zürich, Switzerland, [17]. ^2^ Defined daily doses for animals (DDDvet) and defined course doses for animals (DCDvet): European Surveillance of Veterinary Antimicrobial Consumption (ESVAC), and Revised ESVAC reflection paper on collecting data on consumption of antimicrobial agents per animal species, on technical units of measurement and indicators for reporting consumption of antimicrobial agents in animals [13,14]. ^3^ Number of antimicrobial treatments administered on the respective farm. ^4^ The percentage of the treatment incidence (TI) on the total TI is given for each farm and was calculated using the values of the Swiss Veterinary Medicines Compendium. ^5^ The percentage of the TI on the total TI is given for each farm and was calculated using the values of the European Medicines Agency. ^6^ Sum of respective parameters for all farms; sum for the treatment for which TI_swiss_ and TI_DDD_ are in good agreement (highlighted in grey).

**Table 6:** Antimicrobial drugs for oral and parenteral use and their mean daily dose calculated from detailed field treatment data of 38 Swiss veal operations (*n* = 1854 treatments) and from the summary of product characteristics of the Swiss Veterinary Medicines Compendium ^1^. These hypothetical ‘Swiss’ daily doses were obtained by solving the European Medicines Agency’s equation for treatment incidence ^2^ calculation for the position of the factor of defined daily doses (DDD) and integrating all remaining variables from field treatment data.

Administration Route	Drug	Antimicrobial Class	n ^3^	Mean Daily Dose	DDD
Oral Use	Tylosin	Macrolides	218	6.09	41
	Sulfadimidin	Sulfonamides	221	34.17	105
	Spiramycine	Macrolides	91	14.21	35
	Sulfadimidin *	Sulfonamides	55	14.6	30
	Phthalylsulfathiazole *	Sulfonamides	56	14.74	25
	Chlortetracycline	Tetracyclines	309	21.35	22
	Amoxicillin	Penicillins	197	20.72	20
	Trimethoprim *	Diaminopyrimidins	55	5.84	4.8
	Doxycycline	Tetracyclines	77	12.92	10
Parenteral Use	Tilmicosin	Macrolides	13	2.45	4
	Dehydrostreptomycin	Aminoglycosides	30	15.5	25
	Marbofloxacin	Fluoroquinolones	31	2.66	3.6
	Tulathromycin	Macrolides	46	0.25	0.3
	Amoxicillin	Penicillins	26	7.17	8.3
	Oxytetracycline	Tetracyclines	207	5.72	6.5
	Florfenicol	Florfenicols	101	11.68	13
	Danofloxacin	Fluoroquinolones	19	2.59	1.9

^1^ Tierarzneimittelkompendium der Schweiz, Institute of Pharmacology and Toxicology, Vetsuisse Faculty Zürich, Switzerland, [17]. ^2^ Defined daily doses for animals (DDDvet) and defined course doses for animals (DCDvet): European Surveillance of Veterinary Antimicrobial Consumption (ESVAC), and Revised ESVAC reflection paper on collecting data on consumption of antimicrobial agents per animal species, on technical units of measurement and indicators for reporting consumption of antimicrobial agents in animals [13,14]. ^3^ Number of antimicrobial treatments containing the respective drug. * In combination with trimethoprim or a sulfonamide, respectively.

## Data Availability

The data presented in this study are available on request.
